# ^18^ F-fluoro-deoxy-glucose focal uptake in very small pulmonary nodules: fact or artifact? Case reports

**DOI:** 10.1186/1477-7819-10-71

**Published:** 2012-04-28

**Authors:** Maria Lucia Calcagni, Silvia Taralli, Fabio Maggi, Vittoria Rufini, Giorgio Treglia, Lucia Leccisotti, Lorenzo Bonomo, Alessandro Giordano

**Affiliations:** 1 Institute of Nuclear Medicine, Università Cattolica del Sacro Cuore, Largo A. Gemelli, 8, 00168, Rome, Italy; 2 Institute of Radiology, Università Cattolica del Sacro Cuore, Rome, Italy

**Keywords:** PET-CT, ^18^ F-FDG focal uptake, Small pulmonary nodules

## Abstract

**Background:**

^18^F-fluoro-deoxy-glucose (^18^ F-FDG) positron emission tomography integrated/combined with computed tomography (PET-CT) provides the best diagnostic results in the metabolic characterization of undetermined solid pulmonary nodules. The diagnostic performance of ^18^ F-FDG is similar for nodules measuring at least 1 cm and for larger masses, but few data exist for nodules smaller than 1 cm.

**Case presentation:**

We report five cases of oncologic patients showing focal lung ^18^ F-FDG uptake on PET-CT in nodules smaller than 1 cm. We also discuss the most common causes of ^18^ F-FDG false-positive and false-negative results in the pulmonary parenchyma.

In patient 1, contrast-enhanced CT performed 10 days before PET-CT did not show any abnormality in the site of uptake; in patient 2, high-resolution CT performed 1 month after PET showed a bronchiole filled with dense material interpreted as a mucoid impaction; in patient 3, contrast-enhanced CT performed 15 days before PET-CT did not identify any nodules; in patients 4 and 5, contrast-enhanced CT revealed a nodule smaller than 1 cm which could not be characterized. The ^18^ F-FDG uptake at follow-up confirmed the malignant nature of pulmonary nodules smaller than 1 cm which were undetectable, misinterpreted, not recognized or undetermined at contrast-enhanced CT.

**Conclusion:**

In all five oncologic patients, ^18^ F-FDG was able to metabolically characterize as malignant those nodules smaller than 1 cm, underlining that: ^18^ F-FDG uptake is not only a function of tumor size but it is strongly related to the tumor biology; functional alterations may precede morphologic abnormalities. In the oncologic population, especially in higher-risk patients, PET can be performed even when the nodules are smaller than 1 cm, because it might give an earlier characterization and, sometimes, could guide in the identification of alterations missed on CT.

## Background

The metabolic characterization of undetermined solid pulmonary nodules detected at computed tomography (CT) is one of the first indications for ^18^ F-fluoro-deoxy-glucose (^18^F-FDG) positron emission tomography (PET) [1]. In this setting, integrated ^18^ F-FDG PET-CT provides higher values of sensitivity, specificity, and accuracy (97%, 85%, 93%, respectively) when compared with ^18^ F-FDG PET alone [2]. The diagnostic performance of ^18^ F-FDG is similar for nodules measuring at least 1 cm and for larger masses, but few data exist on its diagnostic performance for nodules smaller than 1 cm [3]. The few false-negative ^18^ F-FDG results can be related to ‘metabolic’ causes such as low ^18^ F-FDG avidity, or to ‘technical’ aspects, commonly related to the nodule size [2]. Likewise, false-positive ^18^ F-FDG results can be related to ‘metabolic’ causes such as inflammatory processes, or to ‘technical’ aspects such as micro-embolisms provoked during the tracer injection.

Herein we report on five cases of focal lung ^18^ F-FDG uptake in pulmonary nodules smaller than 1 cm, and we briefly discuss the most common causes of ^18^ F-FDG false-positive and false-negative results in the pulmonary parenchyma.

## Case presentation

### Case 1: ‘the undetectable nodule’

**Figure 1:**
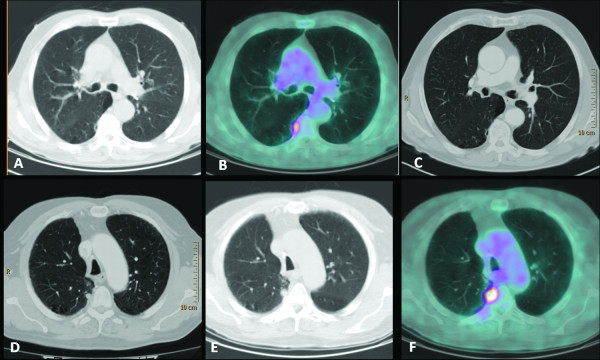
**The undetectable nodule. **^18^ F-FDG PET-CT (June 2010): CT (**a**) and fused (**b**) axial images. Focal ^18^ F-FDG uptake is evident in the subpleural parenchyma of the apical segment of the right lower lobe. (**c**) Contrast-enhanced CT axial image (June 2010): no significant alterations in the site of ^18^F -FDG uptake. (**d**) Contrast-enhanced CT axial image (January 2011): a pulmonary nodule of 1 cm in the apical segment of the right lower lobe, corresponding to the site of ^18^F -FDG uptake is revealed. ^18^ F-FDG PET-CT (April 2011): CT (**e**) and fused (**f**) axial images. Clear evidence in the apical segment of the right lower lobe of a pulmonary nodule characterized by intense metabolic activity.

A 72-year-old male patient, current smoker, with no oncological history, underwent whole-body contrast-enhanced CT in June 2010 because of persistent cough and dyspnoea. CT revealed a mass in the upper right lobe of the lung and the biopsy proved a squamous cell carcinoma. PET-CT performed 1 week later for staging showed intense ^18^ F-FDG uptake in the pulmonary mass with a central photopenic area (SUV maximum 11.6). In addition, there was a focus of ^18^ F-FDG activity localized in the subpleural parenchyma of the apical segment of the lower right lobe, with no significant alteration at the co-registered unenhanced CT images (Figure 1a and b). A technical aspect responsible for an ^18^ F-FDG artifact (micro-embolism due to the tracer injection) was excluded; the ^18^ F-FDG activity was considered non-specific also due to the absence of any suspicious morphological findings at the whole-body contrast-enhanced CT (Figure 1c). After surgeon consultation, in August 2010 the patient underwent an upper right lobectomy with regional lymphoadenectomy: histology confirmed a moderately differentiated (G2) squamous cell carcinoma with large necrotic areas and no lymph node metastases (pT2bN0). A whole-body contrast-enhanced CT performed 4 months later for re-evaluation revealed a pulmonary nodule of 1 cm located in the apical segment of the lower right lobe, suspicious for malignancy (Figure 1d). PET-CT was required for characterization: the scan showed intense ^18^ F-FDG uptake (SUV maximum 10) in the nodule, corroborating its malignancy. It was located in exactly the same area as the focus of ^18^ F-FDG activity at the previous PET-CT (Figure 1e and f). CT-guided biopsy was attempted but it failed because the technical limits due to the interposition of the arch rib and the transverse process. Taking into account all clinical and diagnostic imaging results, the oncologists considered the nodule of neoplastic nature and stereotactic radiotherapy was planned.

### Case 2: ‘the misinterpreted nodule’

**Figure 2:**
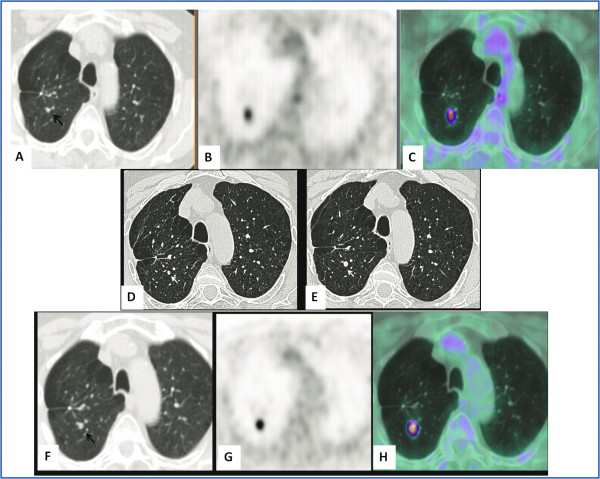
**The misinterpreted nodule. **^18^ F-FDG PET-CT (June 2009): CT (**a**), PET (**b**), and fused (**c**) axial images. Focal ^18^ F-FDG uptake is evident in the posterior segment of the upper right lobe of the lung, projecting onto a vascular bifurcation of peripheral lung vessels (black arrow). (**d**) Axial high-resolution CT image (July 2009): a rounded image interpreted as a mucoid impaction (white arrow) is evident in the posterior segment of the upper right lobe. (**e**) Axial high-resolution CT image (October 2009): a 7 mm pulmonary nodule corresponding to the site of ^18^ F -FDG uptake (white arrow) is revealed. ^18^ F-FDG PET-CT (November 2009): CT (**f**), PET (**g**) and fused (**h**) axial images. Clear evidence of a pulmonary nodule (black arrow) characterized by intense metabolic activity in the posterior segment of the upper right lobe.

A 69-year-old male patient, a long-time smoker (20 pack-years smoking history), presented with chronic obstructive pulmonary disease (COPD) and a history of three different cancers: laryngeal cancer treated with laryngectomy in 1990; pulmonary adenocarcinoma (pT1; G2) treated with wedge resection of the upper right lobe and adjuvant chemotherapy in 2007; and prostate cancer treated with radiation therapy in 2008. ^18^ F-FDG PET-CT and whole-body CT for follow-up in 2008 did not show any abnormal finding; ^18^ F-FDG PET-CT performed in June 2009 showed focal ^18^ F-FDG uptake (SUV maximum 1.9) in the posterior segment of the upper right lobe of the lung, projecting onto a vascular bifurcation of peripheral lung vessels (Figure 2a, b, and c). The focal ^18^ F-FDG uptake did not correspond to any visible anatomical abnormality in the lung parenchyma at the co-registered unenhanced CT, or at the contrast-enhanced CT performed 9 months earlier. A technical aspect responsible for an ^18^ F-FDG artifact was excluded. Therefore, to clarify the nature of this PET finding, taking into account the history and the high oncological risk of the patient, high-resolution chest CT was advised after 3 months. A first high-resolution chest CT performed in July 2009, showed a bronchiole filled with dense material at the site of ^18^ F-FDG uptake (Figure 2d). This was interpreted as a mucoid impaction; a second high-resolution chest CT, performed in October 2009, revealed a growth of dense endobronchial material with nodular morphology (7 mm) in the posterior segment of the upper right lobe (Figure 2e). The nodule corresponded exactly to the focal ^18^ F-FDG uptake revealed at PET-CT performed 4 months earlier and it was considered highly suspicious for malignancy. Before the planned surgery, ^18^ F-FDG PET-CT was repeated, confirming intense metabolic activity (SUV maximum 7.7) only in the pulmonary nodule (Figure 2f, g, and h). The patient underwent an upper right lobectomy and histology revealed an undifferentiated carcinoma with morphological and immunological aspects of a high grade neuroendocrine carcinoma with a high mitotic index and multiple necrotic areas.

### Case 3: ‘the not recognized nodule’

**Figure 3:**
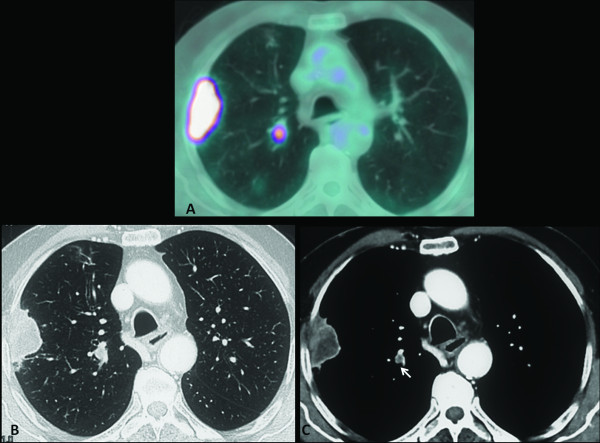
**The not recognized nodule.** (**a**) ^18^ F-FDG PET-CT fused axial image: intense ^18^ F-FDG uptake in the mass of the upper right lobe. Another focus of intense ^18^ F-FDG uptake was evident medially in the upper right lobe. (**b, c**) A retrospective analysis of contrast-enhanced CT revealed a small nodule located near vessels corresponding to the site of ^18^ F-FDG uptake (white arrow).

An 85-year-old male patient (40 pack-years smoking history, and a non-smoker for the last 25 years) with no oncological history, underwent whole-body contrast-enhanced CT in March 2010, because of persistent haemoptysis and dyspnoea. CT revealed a soft tissue subpleural mass in the upper right lobe of the lung and biopsy proved an adenocarcinoma, G3. ^18^ F-FDG PET-CT, performed 2 weeks later for staging, showed intense ^18^ F-FDG uptake in the known mass of the upper right lobe. In addition, there was a focus of intense ^18^ F-FDG uptake in the lung parenchyma of the upper right lobe, projecting onto the branch vessels, while no nodule was clearly shown at the co-registered unenhanced CT (Figure 3a). A second reading of the contrast-enhanced CT, guided by the PET finding, recognized a small (6 mm) juxtavascular nodule at the site of the increased ^18^ F-FDG uptake (Figure 3b and c). Its neoplastic nature was confirmed by the subsequent response to radiation therapy. Indeed, whole-body CT performed in September at the end of treatment, revealed a decrease in size of the upper lobe mass, the disappearance of the upper right lobe nodule, and a concomitant bilateral pulmonary and abdominal-pelvic progression of the disease.

### Case 4: ‘the undetermined nodule’

**Figure 4:**
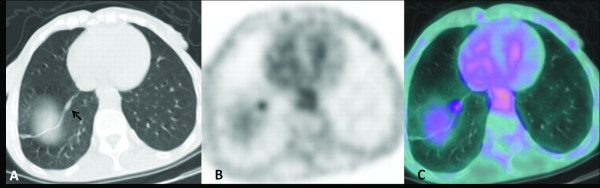
**The undetermined nodule. **^18^ F-FDG PET-CT: CT (**a**), PET (**b**)**,** and fused (**c**) axial images. A focus of intense ^18^ F-FDG uptake was evident in the lower right lobe corresponding to the nodule located in the context of an atelectatic band (black arrow).

A 70-year-old female patient presented with a history of pancreatic cancer diagnosed in August 2008 and treated with duodeno-cephalo-pancreasectomy and adjuvant chemotherapy. For a re-evaluation, whole-body contrast-enhanced CT was performed in January 2010. The CT showed an abdominal recurrence of disease and a pulmonary nodule of 8 mm located in the medial basal segment of the lower right lobe of the lung, in the context of an atelectatic band. Since it was not present at a previous contrast-enhanced CT performed in January 2009, it was considered suspect for malignancy, but it could not be characterized because of its small size and the presence of atelectasis. A new chest contrast-enhanced CT was advised after 3 months, but instead of CT, an ^18^ F-FDG PET-CT was performed in March 2010 for restaging. PET-CT showed intense ^18^ F-FDG uptake in a solid abdominal mass anterior to the abdominal aorta, and focal ^18^ F-FDG uptake in the known 8 mm pulmonary nodule located in the lower right lobe, in the context of atelectasis (Figure 4a, b, and c). After two cycles of chemotherapy, in June 2010, the patient died because of tumor progression.

### Case 5: ‘the smallest nodule’

**Figure 5:**
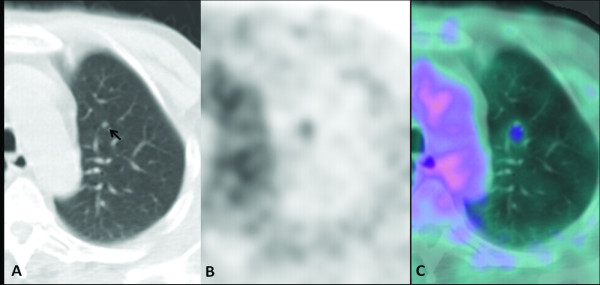
**The smallest nodule. **^18^ F-FDG PET-CT: CT (**a**), PET (**b**)**,** and fused (**c**) axial images. A focal ^18^ F-FDG uptake was evident in a very small nodule (black arrow) located in the upper left lobe.

A 76-year-old male patient presented with a history of Hurthle cell thyroid carcinoma treated with total thyroidectomy in 2003 followed by ^131^I-radiometabolic therapy. In 2008 he underwent a second cycle of ^131^I-radiometabolic therapy for high levels of thyroglobulin (Tg) with no evidence of recurrence or metastases at ^131^I whole-body scintigraphy. Because of progressive increase of Tg values under suppressive hormone therapy (latest value 6 ng/mL), in April 2010 the patient underwent ^18^ F-FDG PET-CT in hypothyroid conditions for re-staging. The study showed a focal ^18^ F-FDG uptake in a pulmonary micronodule of 4 mm located in the upper left lobe, compatible with micro-metastasis (Figure 5a, b, and c). In November 2010 the Tg value under suppressive hormone therapy increased to 9 ng/mL, and high-resolution CT showed an increase in size of the nodule and multiple bilateral pulmonary nodules, suggesting disease progression. The clinicians decided to perform another ^131^I-radiometabolic therapy.

## Discussion

Contrast-enhanced CT is the best diagnostic technique to detect pulmonary nodules providing anatomic and morphologic information [2]. One of the well-established indications for ^18^ F-FDG PET-CT is the metabolic characterization of undetermined pulmonary nodules either in oncologic patients or in patients without any known malignancy but with a high risk of lung cancer [4]. The complementary roles of anatomic and metabolic imaging improve the diagnostic accuracy in the diagnosis and characterization of pulmonary nodules [2]. The risk of malignancy in pulmonary nodules, especially in those smaller than 1 cm, depends on several factors related to the patient, such as a clinical history of previous malignancy, age, smoking history, and on radiological criteria such as nodule size, margins, and density [5-7].

The diagnostic performance of ^18^ F-FDG is similar for nodules measuring at least 1 cm and for larger masses with a sensitivity of 96% and specificity of 73% [3]. Unfortunately, few data exist on the diagnostic performance of ^18^ F-FDG for nodules smaller than 1 cm and the relationship with the risk of malignancy. In the oncologic population, Reinhardt *et al*. [8] report a PET sensitivity of 40% and 78% in pulmonary metastases ranging from 5–7 mm and 8–10 mm, respectively. In both the oncologic and non-oncologic populations, Herder *et al.*[9] report a PET sensitivity of 93% and specificity of 77% in undetermined pulmonary nodules ≤10 mm; in the non-oncologic high risk population, Kim *et al*. [2] report a sensitivity of 50% in nodules ranging from 7–10 mm and Divisi *et al*. [10] report a sensitivity of 95% in solitary lung nodules between 5 and 9.9 mm. ^18^ F-FDG PET sensitivity for characterizing pulmonary nodules as probably malignant can be compromised by ‘metabolic’ causes and ‘technical’ aspects, which lead to false-negative results.

The metabolic causes which can reduce sensitivity are: (1) a high blood glucose level, because of competitive reaction [11]; (2) ^18^ F-FDG avidity related to the histological pattern: low in some tumor types and in slow-growing tumors (bronchiolo-alveolar carcinoma, carcinoid, metastasis of clear-cell renal-cell carcinoma, etc.) [12]; (3) the ^18^ F-FDG uptake related to the degree of cell differentiation: lower in well-differentiated cells than in moderately differentiated ones [13]; (4) the number of viable malignant cells. Fischer *et al*. [14] demonstrated ‘in vitro’ that the theoretical detection limit of ^18^ F-FDG is in the magnitude of 10^5^ to 10^6^ malignant cells, depending on the glucose turnover of the specific cancer. Recently, Wahl *et al*. [15] reported ‘in vivo’ that the limit of ^18^ F-FDG PET for detecting cancers is generally in the magnitude of 10^8^-10^9^ cells, which translates into a tumor size between 0.4 and 1 cm in diameter.

The technical aspects that can determine an underestimation of the true ^18^ F-FDG activity, especially in nodules smaller than 1 cm, are: the respiratory motion because of the displacement caused by shallow breathing, particularly in nodules located in the periphery and in the base of the lungs; the partial volume effect because nodules smaller than the resolution of the PET scanners (ranging from 6 to 10 mm in clinical applications) are not, or only faintly visualized [8,16,17]. Likewise, ^18^ F-FDG PET specificity for characterizing pulmonary nodules as probably malignant can be compromised by ‘metabolic’ causes and ‘technical’ aspects, which lead to false-positive results.

The metabolic causes are: benign neoplasms (sclerosis hemangioma, leiomyoma, and so on), infection (tuberculosis, sarcoidosis, and so on) or inflammation (acute inflammation associated with bronchiectasis or thromboembolic disease, and so on) [12,18-20]. To our knowledge, a pulmonary micro-embolism provoked during ^18^ F-FDG injection can be considered the only reason ‘technically’ responsible for a false-positive result. The vascular endothelium can be damaged by several factors, such as a para-venous or ‘in bolo’ injection, producing micro-emboli. The cellular activation process at the site of pulmonary micro-emboli requires energy with a consequent increased glucose uptake [21,22]. From a ‘scintigraphic’ point of view, this is evident as a focal ^18^ F-FDG lung uptake but, thanks to CT integrated with the PET scanner, its artifactual nature should be suspected in the absence of corresponding abnormalities at the co-registered CT [23].

Our first case showed focal ^18^ F-FDG activity in the lung parenchyma in the absence of any detectable abnormality, even at co-registered unenhanced CT. We could exclude an artifact caused by the micro-embolism provoked during injection because: we routinely inject the radiotracers through a venous cannula avoiding a para-venous injection and repeated blood aspirations; no bolus injection was performed and no vascular activity due to a para-venous injection in the arm was evident in the images. The focus of ^18^ F-FDG activity projecting onto the subpleural parenchyma was considered as non-specific, also because at contrast-enhanced CT performed only ten days before, no other morphological abnormalities were detectable except the known mass. It is very interesting to note that the ^18^ F-FDG activity was already evident 7 months before the CT appearance of a 1 cm nodule, therefore indicating its malignant nature. The absence of a detectable nodule at the first CT examination suggests that the area of focal ^18^ F-FDG uptake contained a very small number of tumor cells, not enough for anatomical detection, and therefore, it would be reasonably to say around the lowest ^18^ F-FDG detection limit, as reported [15]. This supports the well-known concept that the metabolic/functional alterations may precede the morphologic ones, and PET can sometimes detect early changes not, or only minimally revealed by morphological imaging [24].

Also in case 2 we could exclude an artifact due to a micro-embolism by applying the same considerations previously described. Differently from case 1, taking into account the very high oncologic risk of this patient, the PET finding was considered highly suspicious for malignancy, even in the absence of any clear morphologic abnormalities at co-registered unenhanced CT. No clear evidence of a nodule at co-registered unenhanced CT could be explained by some technical acquisition reasons: slice thickness (5 mm), free breathing and ‘low dose’ setting (tube current of about 40 mA/s). These acquisition parameters could limit the detection of small pulmonary nodules at co-registered unenhanced PET-CT, especially in a juxtavascular location. A volumetric high-resolution CT was suggested to overcome these limits; with this technique, slice thickness is 1 mm or less, in a single, breath-held inspiration, with a full radiation dose, allowing the identification of small pulmonary nodules. However, a delay of 3 months was suggested to identify dimensional growth, as this is the only reliable criterion in favor of malignancy; the other criteria commonly used to define malignancy of a nodule (dimensions, site, margins) are less reliable in small nodules [25-27]. Also in this case, PET was able to detect malignant findings earlier than morphological imaging. No clear detection of the nodule at co-registered unenhanced CT and also at the first high-resolution CT suggests that the number of tumor cells showing ^18^ F-FDG uptake was very low, as previously reported for case 1 [15]. In addition, very early ^18^ F-FDG uptake could be explained by the high mitotic index and by the presence of highly aggressive and undifferentiated cells, as proved by histopathology.

In case 3, PET showed an intense focal ^18^ F-FDG uptake in the right lung, located between two vessels, where no abnormalities were recognized either at the staging contrast-enhanced CT, or at CT integrated with PET. Also in this case we could exclude the ‘technical’ origin of a micro-embolism. So, guided by focal ^18^ F-FDG uptake, we carefully looked for any alteration in lung parenchyma at co-registered unenhanced CT as well as at contrast-enhanced CT. Eventually, a small juxtavascular nodule corresponding to the focal uptake was recognized, and its neoplastic nature was confirmed by the subsequent response to radiation therapy, even if a disease progression with multiple new pulmonary nodules and neoplastic abdominal disease were found. Possible causes of failed identification of pulmonary nodules at CT are a central location, either within the bronchi (as in case 2) or adjacent to vessels, as in this case; other possible causes are a small size, faint attenuation, lower lobe location or location adjacent to other parenchymal abnormalities such as inflammatory lesions [28]. This case underlines the importance of looking at the pulmonary parenchyma with high accuracy, especially in high-risk oncological patients, searching for any abnormality and eventually using PET findings as a guide because PET could sometimes guide the identification of alterations missed at morphologic imaging.

In case 4, CT revealed a pulmonary nodule not present in the previous CT scan, difficult to be characterized because of its small size and the presence of the atelectatic band. PET performed only 1 month later demonstrated that the nodule had metabolic activity, strongly suggesting its neoplastic nature. Unfortunately, the patient died for cancer progression and no proof of its true nature was available. Multidetector CT can detect nodules as small as 1 or 2 mm but without being able to characterize them. Nevertheless, in this case, the pulmonary nodule was characterized by PET even though located at the base of right lung, in the context of the atelectatic band and only 8 mm in size.

In case 5, PET was able to characterize a pulmonary micronodule of 4 mm, impossible to characterize at morphologic imaging. Despite the very small size, the ^18^ F-FDG uptake can be explained by ‘metabolic’ reasons such as the aggressive histotype of the primary thyroid cancer (Hurthle cell carcinoma), and the de-differentiation of the tumor cells, rather than their number. It has been widely demonstrated that secondary lesions of thyroid carcinomas with low iodine avidity tend to have higher glucose metabolism and are more likely to be positive at ^18^ F-FDG PET [29].

## Conclusions

In conclusion, ^18^ F-FDG was able to metabolically characterize as malignant nodules smaller than 1 cm, underlining, although in only five oncologic patients, the following concepts: (1) ^18^ F-FDG uptake is not only a function of tumor size but it is strongly related to the tumor biology, such as mitotic index, aggressivity, differentiation grading, de-differentation of the cells; and (2) our five cases further confirm the general principle that ‘functional alterations may precede morphologic abnormalities’.

In clinical practice, in the oncologic population, especially in higher-risk patients (such as those with multiple tumors, with a history of recurrence, and/or with an aggressive histotype), PET can be performed even when the nodules are smaller than 1 cm, because it might give an earlier characterization. Moreover, PET could sometimes guide the identification of alterations missed at morphologic imaging. It is reasonable to assume that the additional radiological exposure risk (effective dose of 7–10 mSv) can be ‘balanced’ by the possibility of early diagnosis and treatment of an aggressive tumor [24].

## Abbreviations

18 F-FDG: 18F-fluoro-deoxy-glucose; CT: Computed tomography; PET: Positron emission tomography; PET-CT: Positron emission tomography-computed tomography; SUV: (standardized uptake value).

## Competing interests

The authors declare that they have no competing interests.

## Authors’ contributions

MLC and ST drafted the manuscript, contributed to conception, manuscript and design preparation, and conducted a literature search. MLC, ST, FM, VR, and LB performed and analyzed PET and CT images. LL, GT, and FM contributed to acquisition of data and obtained images used in the manuscript. VR participated in the design of the study. LB and AG critically revised the manuscript. All authors read and approved the final manuscript.

## Consent

Written informed consent was obtained from the patients for publication of this case report and any accompanying images. A copy of the written consent is available for review by the Editor-in-Chief of this journal.
